# Distribution and prognostic impact of *EGFR* and *KRAS* mutations according to histological subtype and tumor invasion status in pTis-3N0M0 lung adenocarcinoma

**DOI:** 10.1186/s12885-023-10716-6

**Published:** 2023-03-14

**Authors:** Masaoki Ito, Yoshihiro Miyata, Kei Kushitani, Daisuke Ueda, Yukio Takeshima, Morihito Okada

**Affiliations:** 1 Department of Surgical Oncology, Research Institute for Radiation Biology and Medicine, Hiroshima University, 1-2-3 Kasumi, Minami-ku, 734-8551 Hiroshima, Japan; 2 Department of Pathology, Graduate School of Biomedical & Health Sciences, Hiroshima University, 1-2-3 Kasumi, Minami-ku, 734-8551 Hiroshima, Japan

**Keywords:** *EGFR*, IASLC, *KRAS*, Lung adenocarcinoma, Prognosis, Recurrence, Staging, Surgery

## Abstract

**Background:**

The prognostic impact of *EGFR mutation* as major targetable somatic gene variant on lung adenocarcinoma is controversial. *KRAS* is another major somatic variant in lung adenocarcinoma, and a therapeutic agent for *KRAS* G12C became available in clinical settings. These mutations represent clinicopathological features of lung adenocarcinoma and can guide the treatment choice after recurrence. We evaluated the prognostic impact of *EGFR* and *KRAS* mutations by considering other clinicopathological recurrence risks in resected pTis-3N0M0 lung adenocarcinoma.

**Methods:**

Clinicopathological features related to recurrence and genetic status were estimated in consecutive 877 resected cases. Recurrence-free survival (RFS), cumulative recurrence rate (CRR), and overall survival (OS) were compared. Uni- and multivariate analyses for RFS were performed after excluding cases with little or no recurrence risks.

**Results:**

*EGFR* mutation was more likely to be harbored in female, never-smoker, or patients accompanied by > 5% lepidic component. *KRAS* mutation was more likely to be harbored in patients with current/ex-smoking history, International Association for the Study of Lung Cancer (IASLC) grade 3, or accompanied lymphatic or vascular invasion. In IASLC grade 2 and 3 patients, *EGFR* or *KRAS* mutation cases had significantly worse 5-year RFS than wild type patients (76.9% vs. 85.0%, hazard ratio [HR] = 1.55, 95% confidence interval [CI] = 1.62–6.41, *P* < 0.001). *EGFR* or *KRAS* mutation cases had significantly higher 5-year CRR than wild type patients (17.7% vs. 9.8%, HR = 1.69, 95% CI = 1.44–6.59, *P* = 0.0038). *KRAS* mutation cases had higher 5-year CRR than *EGFR* mutation cases (16.7% vs. 21.4%, HR = 1.62, 95% CI = 0.96–7.19, *P* = 0.061). There was no significant difference in OS between cohorts. Multivariate analysis revealed that a positive *EGFR*/*KRAS* mutation status was risk factor for worse RFS (HR = 2.007, 95% CI = 1.265–3.183, *P* = 0.003).

**Conclusion:**

Positive *EGFR* and *KRAS* mutation statuses were risk factors for recurrence in resected IASLC grade 2 and 3 patients. *KRAS* mutations were more likely to be confirmed in cases with an increased risk of recurrence. *EGFR* and *KRAS* mutation statuses should be evaluated simultaneously when assessing the risk of recurrence.

## Background

Curative surgical resection is the optimal treatment for primary lung cancer without lymph nodes or distant metastasis. The risk of recurrence mainly depends on the completion of resection, tumor invasion status (tumor invasive size, pleural invasion, and lymphovascular invasion), and histological features. The prognostic impact of *EGFR* mutation as a cancer-driving gene has been discussed but is still controversial [1–5]. Namely, some previous studies have reported that the presence of an *EGFR* mutation is favorable or non-significance for prognosis, whereas others have concluded that a positive *EGFR* mutation status is a risk factor for worse prognosis based on malignant potential characterized by histological or radiological features. As well as *EGFR* mutation, *KRAS* mutation is also major targetable somatic variable in primary lung adenocarcinoma.

Since the ADAURA study revealed that post-operative osimertinib therapy prolongs disease-free survival in resected stage IB–IIIA *EGFR* mutation-positive patients [6], the evaluation of genetic status has gained increasing attention in resected cases. The therapeutic benefit of a KRAS inhibitor has also been reported in advanced lung cancer cases [7]. KRAS inhibitor for G12C variant has been clinically available, and studies on promising drugs targeting other *KRAS* mutation types are ongoing (ClinicalTrials.gov numbers: NCT05382559, NCT04853017, and NCT04678648) [8]. As a major somatic variant with a clinically available inhibitor, the impact of *KRAS* mutation also becomes more warranted to be revealed in surgically treated cases.

Adenocarcinoma, the most common histological type of lung cancer, consists of several subhistological variants with different malignant potentials. The prevalence or prognostic impact of targetable genetic variants also varies according to tumor invasive and subhistological type. Few study evaluated the prognositc impact of these major targetable genetic variants in consideing with non-gentic prognostic clinicopatholgical features in lung adenoacricnoma. Major targetable genetic variants can serve as prognostic factors representative of clinicopathological features and can be directly useful in treatment strategies after recurrence.

Here, we aimed to evaluate the prognostic impact of *EGFR* and *KRAS* mutations by considering the relationship between these mutations and their histological subtype and/or tumor invasion status in resected pTis-3N0M0 lung adenocarcinoma. In addition, by considering *KRAS* mutaion status, we also aimed furhter understanding about the prognostic impact of *EGFR* mutation.

## Patients and methods

### Study design

We retrospectively reviewed pathological Tis-3N0M0 primary lung adenocarcinoma cases resected at Hiroshima University Hospital between January 2007 and December 2019. Clinicopathological information was collected from medical records. For *EGFR* and *KRAS* mutation testing, DNA was extracted from frozen or formalin-fixed paraffin-embedded resected tissues using the QIAamp DNA FFPE Tissue Kit or QIAamp DNA Micro Kit (56404, 56304 Qiagen GmbH, Hilden, Germany). The following cases were excluded: non-primary adenocarcinoma cases; variant types of adenocarcinoma defined by the International Association for the Study of Lung Cancer (IASLC) classification [9]; cases after preoperative chemotherapy/chemoradiotherapy or palliative surgery; cases without complete clinicopathological background, available tissue samples, or any radiological follow-up after resection; cases with new lesions during follow-ups that were difficult to distinguish as second primary or recurrent tumors. pT4 cases were also excluded due to the small number of cases available. The relationship between *EGFR* or *KRAS* mutation and clinicopathological features and the prognostic impacts of these genetic statuses were investigated. Analyses regarding the prognostic impact were performed after cases with little or no risk of recurrence were excluded, as previously described [1, 2].

### Evaluation of clinicopathological features

Staging was determined using the 8th IASLC TNM staging system [10], and pathological diagnosis was performed by two pathologists according to the 2021 WHO classification [11]. Pathological tumor invasion size was measured directly on resected specimens or calculated by invasive ratio on whole tumor size, as recommended [12]. The pathological grading system was determined according to the IASLC proposal [13]. Simultaneous or heterochronic multiple tumors in a single patient were determined as intrapulmonary metastases or independent tumors, as proposed by the IASLC [14, 15]. Tumors diagnosed as independent were handled as primary tumors. In patients with multiple primary tumors, if one tumor relapsed, observations of another non-relapsed tumor were censored at the time of recurrence.

### EGFR and KRAS mutation detection

Twelve *EGFR* somatic variants (G719C/S/A in Exon 18, seven types of deletion in Exon 19, and L858R/L861Q in Exon 21) were detected by peptide nucleic acid (PNA)-locked nucleic acid (LNA) PCR clamp-based detection test or real-time PCR using an affinity probe (for G719A only, IDT, Coralville, USA), as previously described [1, 16]. Seven types of *KRAS* somatic mutations (G12A/C/D/R/S/V and G13D in Exon 2) were detected by droplet digital PCR using the PrimePCR for ddPCR KRAS multi screening Kit (1863506, BIO-RAD, Hercules, USA). Considering that *EGFR* and *KRAS* mutations are mutually exclusive in principle and that *EGFR* mutations are more common in Asians, *EGFR* mutations were screened first, and then the *KRAS* mutation status was examined in *EGFR* wild type cases.

### Statistical analysis

To evaluate the prognostic impact of genetic status, recurrence-free survival (RFS), cumulative recurrence rate (CRR), and overall survival (OS) were calculated using the Kaplan–Meier method and compared using the log-rank test. RFS was defined from the day of operation to the day of recurrence as detected by a radiological device or of death from any cause. CRR was calculated from the day of operation to the day of recurrence as detected using a radiological device. OS was calculated from the day of operation to the day of death from any cause. The significance of frequency was evaluated using the chi-squared or Yates’s chi-squared test. Patient age and pathological invasive size were compared as continuous variables using the Mann–Whitney U test. The prognostic impact of each variable was estimated by uni- and multivariate analyses using the Cox proportional hazards model with a backward stepwise procedure. Significance was defined as a two-tailed *P*-value < 0.05. Statistical analyses and figure creation were performed using SPSS Version 28.0.1 (IBM Corp., Armonk, NY, USA), StatMate V (ATMS Co., Ltd., Tokyo, Japan), and SynergyFinder Version 3.0 [17].

## Results

### Clinicopathological features

We reviewed 947 pN0M0 cases and finally included 877 cases after excluding 70 inadequate cases. The clinicopathological features of the 877 cases are shown in Table 1. Overall, 404 (46.1%) and 98 (11.2%) patients harbored *EGFR* and *KRAS* mutations, respectively. Neither *EGFR* nor *KRAS* mutations were detected in 375 (42.8%) patients. The median follow-up term was 1373 days.

**Table 1:** Clinicopathological features of the 877 reviewed cases

Clinicopathological characteristics	Patients (*N* = 877)
Age, years	
Median (range, interquartile range)	69.0 (31–91, 63–74)
Sex, N (%)	
Male	439 (50.1)
Female	438 (49.9)
Smoking status, N (%)	
Never	452 (51.5)
Current or ex-	425 (48.5)
Surgical procedure, N (%)	
Pneumonectomy	1 (0.1)
Lobectomy	431 (49.1)
Segmentectomy	291 (33.2)
Wedge resection	154 (17.6)
Pathological invasive size, mm	
Median (range, interquartile range)	10.0 (0–65, 4.0–18.0)
Histological subtype, N (%)	
AIS	156 (17.8)
MIA	103 (11.7)
Lepidic predominance	147 (16.8)
Papillary predominance	397 (45.3)
Acinar predominance	24 (2.7)
Micropapillary predominance	17 (1.9)
Solid predominance	33 (3.8)
IASLC histological grade	
G1	145 (16.5)
G2	360 (41.0)
G3	113 (12.9)
Pleural invasion, N (%)	
Pl0	781 (89.1)
Pl1	62 (7.1)
Pl2	20 (2.3)
Pl3	14 (1.6)
Lymphatic invasion, N (%)	
Negative	794 (90.5)
Positive	83 (9.5)
Vascular invasion, N (%)	
Negative	743 (84.7)
Positive	134 (15.3)
Intrapulmonary metastasis, N (%)	
Negative	859 (97.9)
PM1	18 (2.1)
Pathological T status, N (%)	
Tis	156 (17.8)
Tia (mi)	103 (11.7)
T1a	176 (20.1)
T1b	226 (25.8)
T1c	75 (8.6)
T2a	98 (11.2)
T2b	12 (1.4)
T3	31 (3.5)
Genetic status, N (%)	
* EGFR* mutation	404 (46.1)
* KRAS* mutation	98 (11.2)
* EGFR*/*KRAS* wild type	375 (42.8)
Adjuvant chemotherapy, N (%)	
Done	197 (22.5)
None	680 (77.5)
Recurrence, N (%)	
Negative	807 (92.0)
Positive	70 (8.0)

*Abbreviations*: *AIS* Adenocarcinoma in situ, *IASLC* International Association for the Study of Lung Cancer, *MIA* Minimally invasive adenocarcinoma

**Fig. 1:**
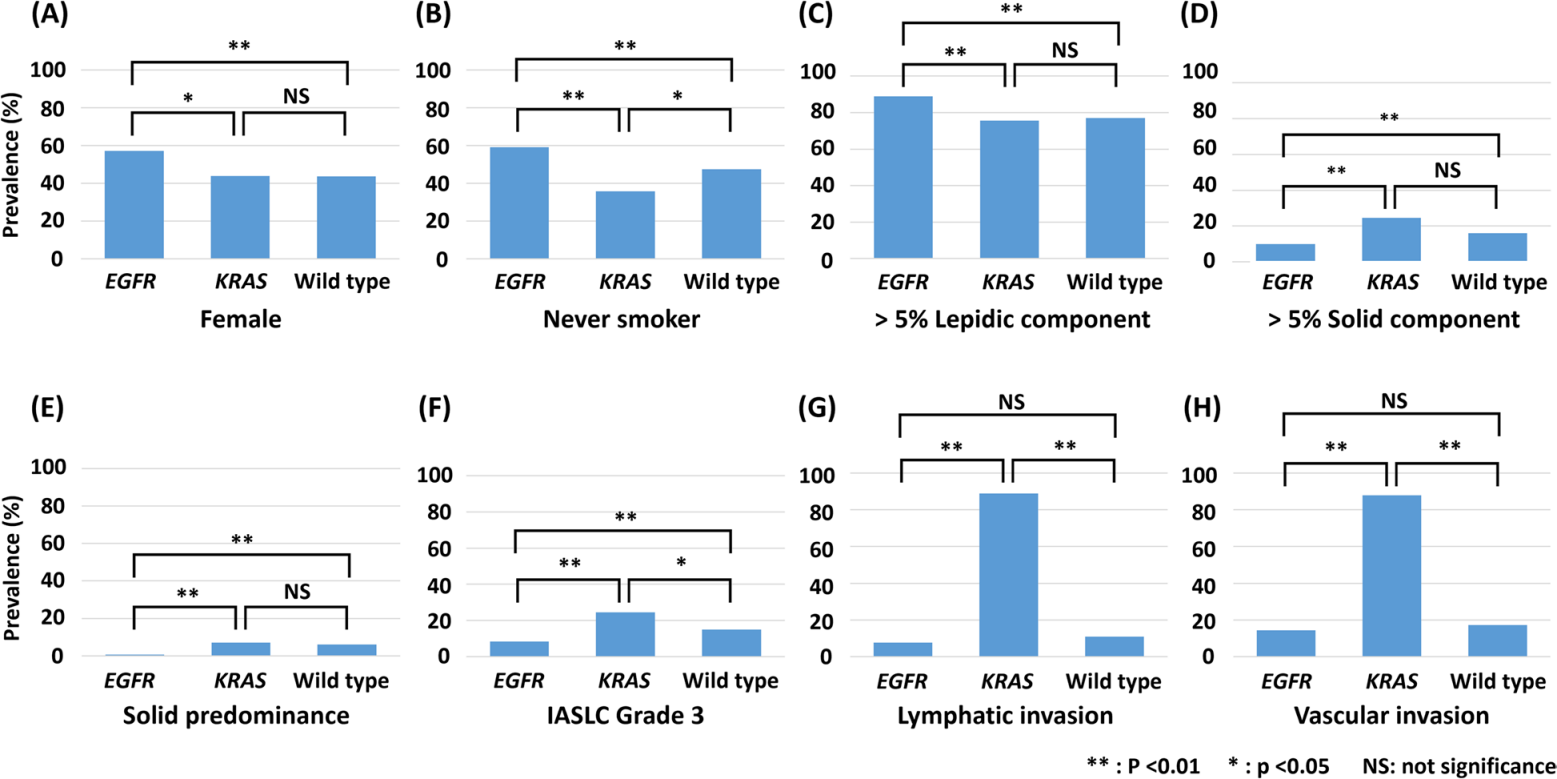
Comparison of prevalence of clinicopathological features according to mutation status. Results from Table 2 were visualized if there were significant differences between groups. **A** Female; **B** never-smoker; **C** > 5% lepidic component; **D** > 5% solid component; **E** solid predominance; **F** IASLC grade 3; **G** lymphatic invasion; **H** vascular invasion. **P < 0.01; *P < 0.05; N.S., not significant. Abbreviations: IASLC, International Association for the Study of Lung Cancer

Genetic status was significantly related to some clinicopathological features (Table 2). Notably, *EGFR* mutations were more likely harbored by females, never-smokers, or patients with > 5% lepidic component (Fig. 1A, B, C) and were less likely to be harbored by patients with > 5% solid component, solid predominant, or IASLC grade 3 than in wild type patients or those harboring *KRAS* mutations (Fig. 1D, E, F). *KRAS* mutations were more likely to be harbored in patients with a current/ex-smoking history, IASLC grade 3, or with lymphatic or vascular invasion than in wild type patients or those harboring *EGFR* mutations (Fig. 1B, F, G, H).

**Table 2:** Comparison of clinicopathological features according to genetic status

Clinicopathological characteristics	Mutant status			*P-*value
	*EGFR (N = 404)*	*KRAS (N = 98)*	Wild type (*N* = 375)	
Age, years				
Median (range, interquartile range)	68 (41–91, 63–75)	70 (49–89, 63–75)	68 (31–89, 61.5–74)	N.S.
Sex, N (%)				
Male	173 (42.8)	55 (56.1)	211 (56.3)	Figure 1
Female	231 (57.2)	43 (43.9)	164 (43.7)	
Smoking status, N (%)				
Never	239 (59.2)	35 (35.7)	178 (47.5)	Figure 1
Current or ex-	165 (40.8)	63 (64.3)	197 (52.5)	
Surgical procedure, N (%)				
Pneumonectomy	0 (0.0)	0 (0.0)	1 (0.3)	
Lobectomy	204 (50.5)	44 (44.9)	183 (48.8)	N.S.
Segmentectomy	135 (33.4)	31 (31.6)	125 (33.3)	N.S.
Wedge resection	65 (16.1)	23 (23.5)	66 (17.6)	N.S.
Pathological invasive size, mm				
Median (range, interquartile range)	10 (0–50, 4–18.25)	11.25 (0–45, 3–16.74)	10.0 (0–65, 3–18)	N.S.
Histological predominance, N (%)				
in situ	65 (16.1)	20 (20.4)	71 (18.9)	N.S.
Minimally invasive	54 (13.4)	10 (10.2)	39 (10.4)	N.S.
Lepidic	71 (17.6)	12 (12.2)	64 (17.1)	N.S.
Papillary	196 (48.5)	43 (43.9)	158 (42.1)	N.S.
Acinar	6 (1.5)	3 (3.1)	15 (4.0)	N.S.
Micropapillary	9 (2.2)	3 (3.1)	5 (1.3)	N.S.
Solid	3 (0.7)	7 (7.1)	23 (6.1)	Figure 1
Accompanied > 5% pathological component, N (%)				
Lepidic	359 (88.9)	74 (75.5)	289 (77.1)	Figure 1
Micropapillary	68 (16.8)	17 (17.3)	61 (16.3)	N.S.
Solid	40 (9.9)	24 (24.5)	60 (16.0)	Figure 1
IASLC histological grade				
G1	69 (17.1)	12 (12.2)	64 (17.1)	N.S.
G2	183 (45.3)	32 (32.7)	145 (38.7)	0.0233^ek^
G3	33 (8.2)	24 (24.5)	56 (14.9)	Figure 1
Pleural invasion, N (%)				
Pl0	369 (91.3)	84 (85.7)	328 (87.5)	N.S.
Pl1	24 (5.9)	12 (12.2)	26 (6.9)	0.0300^ek^
Pl2	8 (2.0)	2 (2.0)	10 (2.7)	N.S.
Pl3	3 (0.7)	0 (0.0)	11 (2.9)	0.0215^ew^
Lymphatic invasion, N (%)				
Negative	373 (92.3)	11 (11.2)	334 (89.1)	Figure 1
Positive	31 (7.7)	87 (88.8)	41 (10.9)	
Vascular invasion, N (%)				
Negative	347 (85.9)	12 (12.2)	310 (82.7)	Figure 1
Positive	57 (14.1)	86 (87.8)	65 (17.3)	
Intrapulmonary metastasis, N (%)				
Negative	400 (99.0)	94 (95.9)	365 (97.3)	0.0283^ek^
PM1	4 (1.0)	4 (4.1)	10 (2.7)	
Pathological T status, N (%)				
Tis	65 (16.1)	20 (20.4)	70 (18.7)	N.S.
T1a (mi)	54 (13.4)	10 (10.2)	39 (10.4)	N.S.
T1a	89 (22.0)	14 (14.3)	74 (19.7)	N.S.
T1b	101 (25.0)	26 (26.5)	99 (26.4)	N.S.
T1c	41 (10.1)	7 (7.1)	27 (7.2)	N.S.
T2a	43 (10.6)	15 (15.3)	40 (10.7)	N.S.
T2b	4 (1.0)	2 (2.0)	6 (1.6)	N.S.
T3	7 (1.7)	4 (4.1)	20 (5.3)	0.0061^ew^
Adjuvant chemotherapy, N (%)				
Done	94 (23.3)	20 (20.4)	83 (22.1)	N.S.
None	310 (76.7)	78 (79.6)	292 (77.9)	
Recurrence, N (%)				
Positive	35 (8.7)	13 (13.3)	22 (5.9)	0.0127^kw^
Negative	369 (91.3)	85 (86.7)	353 (94.1)	

The significance of frequency was estimated between each cohort. *P*-values with “ek,” “kw,” or “ew” indicate significant *P-*values between the *EGFR* mutation and *KRAS* mutation cohort, *KRAS* mutation and wild type cohort, or *EGFR* mutation and wild type cohort, respectively. Where there is significance between two or three combinations, the results are shown in Fig. 1

*Abbreviations*: *IASLC* International Association for the Study of Lung Cancer, *N.S.* No significance between any cohort, *PI* Pleural invasion, *PM* Intrapulmonary metastasis

^*^P < 0.05

### Prognostic impact of histological features

**Fig. 2:**
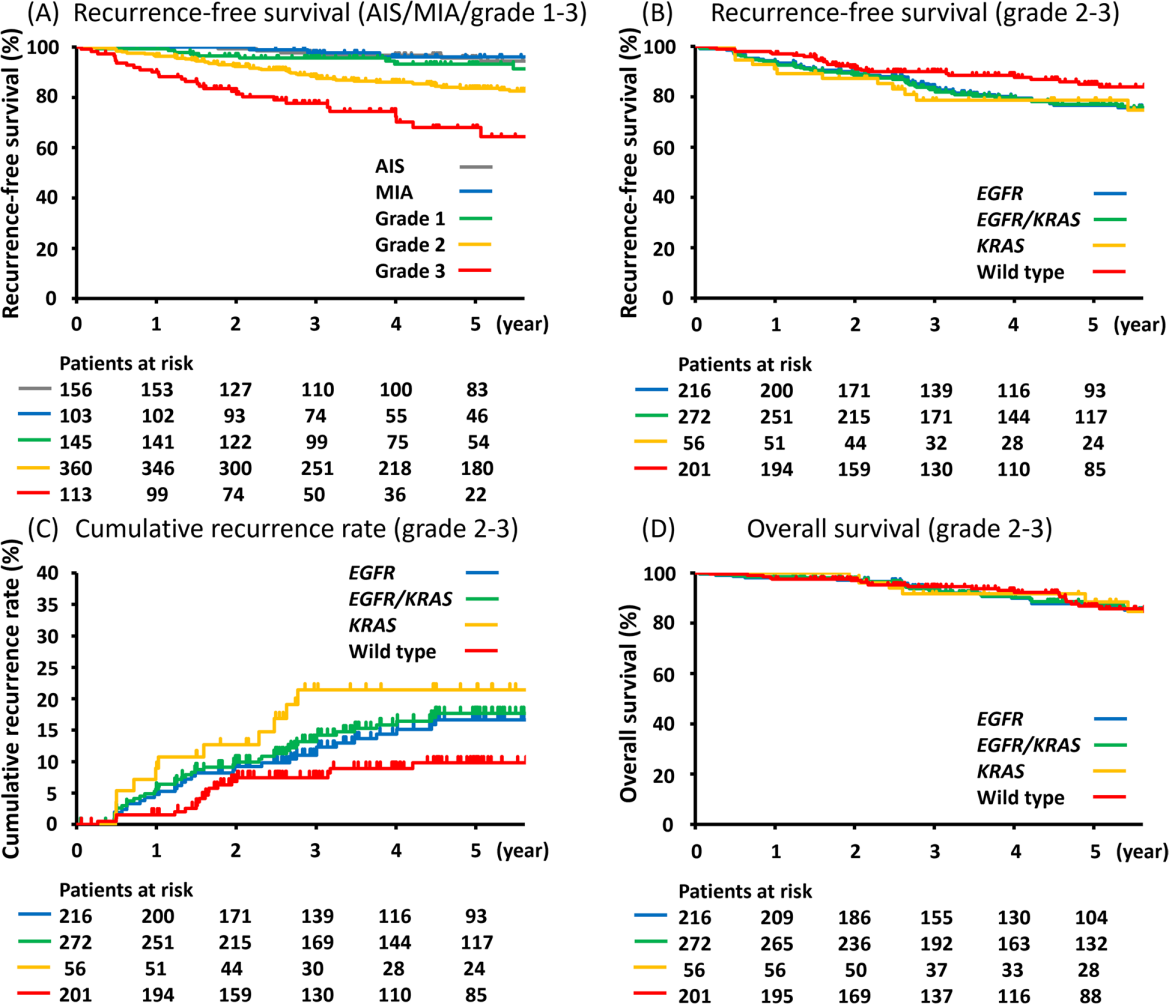
RFS, CRR, or OS curves established using the Kaplan–Meier method. **A** RFS curves by AIS, MIA, and IASLC histological grade. **B** RFS curves by genetic mutation status in IASLC grade 2 and 3 cases. **C** CRR curves by genetic mutation status in IASLC grade 2 and 3 cases. **D** OS curves by genetic mutation status in IASLC grade 2 and 3 cases. Abbreviations: AIS, adenocarcinoma in situ; CRR, cumulative recurrence rate; MIA, minimally invasive adenocarcinoma; OS, overall survival; RFS, recurrence-free survival

Five-year RFS in adenocarcinoma in situ (AIS), minimally invasive adenocarcinoma (MIA), and IASLC grade 1, grade 2, and grade 3 patients was 95.7%, 96.1%, 93.2%, 83.6%, and 68.0%, respectively (Fig. 2A). The recurrence rate in IASLC grade 1 patients was 4.8% (7/145). As previously shown [2, 18, 19], AIS, MIA, and IASLC grade 1 patients had no or little risk of recurrence after complete resection. Thus, the IASLC grading system worked properly in our cohort, and we performed analysis after excluding AIS, MIA, and IASLC grade 1 patients as previously [1, 2].

### Impact of EGFR and KRAS mutation on RFS

In IASLC grade 2 and 3 patients, 5-year RFS in wild type patients and those harboring *EGFR* and *KRAS* mutations was 85.0%, 76.6%, and 78.6%, respectively (Fig. 2B). There was no significant difference in RFS between patients harboring *EGFR* and *KRAS* mutations (hazard ratio [HR] = 0.78, 95% confidence interval [CI] = 0.22–1.39, *P* = 0.209). Five-year RFS in patients harboring *EGFR* or *KRAS* mutations was 76.9%, significantly worse compared to the RFS in wild type patients (HR = 1.55, 95% CI = 1.62–6.41, *P* < 0.001).

### Impact of EGFR and KRAS mutations on CRR

In IASLC grade 2 and 3 patients, 5-year CRR in wild type patients and those harboring *EGFR* and *KRAS* mutations was 9.8%, 16.7%, and 21.4%, respectively (Fig. 2C). In comparison to that in patients harboring *EGFR* mutations, 5-year CRR in patients harboring *KRAS* mutations was higher, but not significantly different (HR = 1.62, 95% CI = 0.96–7.19, *P* = 0.061). The 5-year CRR in patients harboring *EGFR* or *KRAS* mutations was 17.7%, significantly higher than in wild type patients (HR = 1.69, 95% CI = 1.44–6.59, *P* = 0.0038).

### Impact of EGFR and KRAS mutations on OS

In IASLC grade 2 and 3 patients, 5-year OS in wild type patients and those harboring *EGFR* and *KRAS* mutations was 86.8%, and 87.1%, 88.5%, respectively (Fig. 2D). There was no significant difference between any cohorts.

### Uni- and multivariate analyses for RFS

The univariate analysis showed that patient age, current/ex-smoking habits, pathological invasive size, IASLC grade 3, pleural invasion, lymphovascular invasion, intrapulmonary metastasis, and positive *EGFR*/*KRAS* mutation status were significantly related to worse RFS. The multivariate analysis revealed that patient age, pathological invasive size, pleural invasion, lymphovascular invasion, intrapulmonary metastasis, and positive *EGFR*/*KRAS* mutation status were risks for worse RFS (*EGFR*/*KRAS* mutation: HR = 2.007, 95% CI = 1.265–3.183, *P* = 0.003; Table 3).

**Table 3:** Uni- and multivariate analyses for RFS in IASLC grade 2–3 patients (*N* = 473)

	Univariate analysis		Multivariate analysis	
Variable	HR (95% CI)	*P-*value	HR (95% CI)	*P-*value
Sex (male)	1.036 (0.685–1.566)	0.866	0.606 (0.324–1.135)	0.606
Age	1.077 (1.050–1.105)	< 0.001	1.079 (1.051–1.107)	< 0.001
Smoking status (current or ex-)	1.628 (1.062–2.496)	0.025	1.516 (0.965–2.380)	0.071
Surgical procedure (wedge resection)	1.774 (0.984–3.199)	0.057	1.346 (0.718–2.524)	0.354
Pathological invasive size (mm)	1.051 (1.033–1.071)	< 0.001	1.031 (1.009–1.053)	0.005
IASLC grade (grade 3)	2.171 (1.397–3.374)	< 0.001	1.210 (0.733–1.997)	0.456
Pleural invasion (positive)	3.799 (2.491–5.793)	< 0.001	2.784 (1.714–4.520)	< 0.001
Lymphovascular invasion (positive)	2.712 (1.797–4.092)	< 0.001	1.996 (1.227–3.245)	0.005
Intrapulmonary metastasis (positive)	3.644 (1.678–7.914)	0.001	3.845 (1.702–8.687)	0.001
*EGFR*/*KRAS* mutation (positive)	1.553 (1.004–2.402)	0.048	2.007 (1.265–3.183)	0.003

*Abbreviations*: *CI* Confidence interval, *HR* Hazard ratio, *IASLC* International Association for the Study of Lung Cancer

### Distribution of EGFR and KRAS mutant cases according to histological subtype and tumor invasion status

**Fig. 3:**
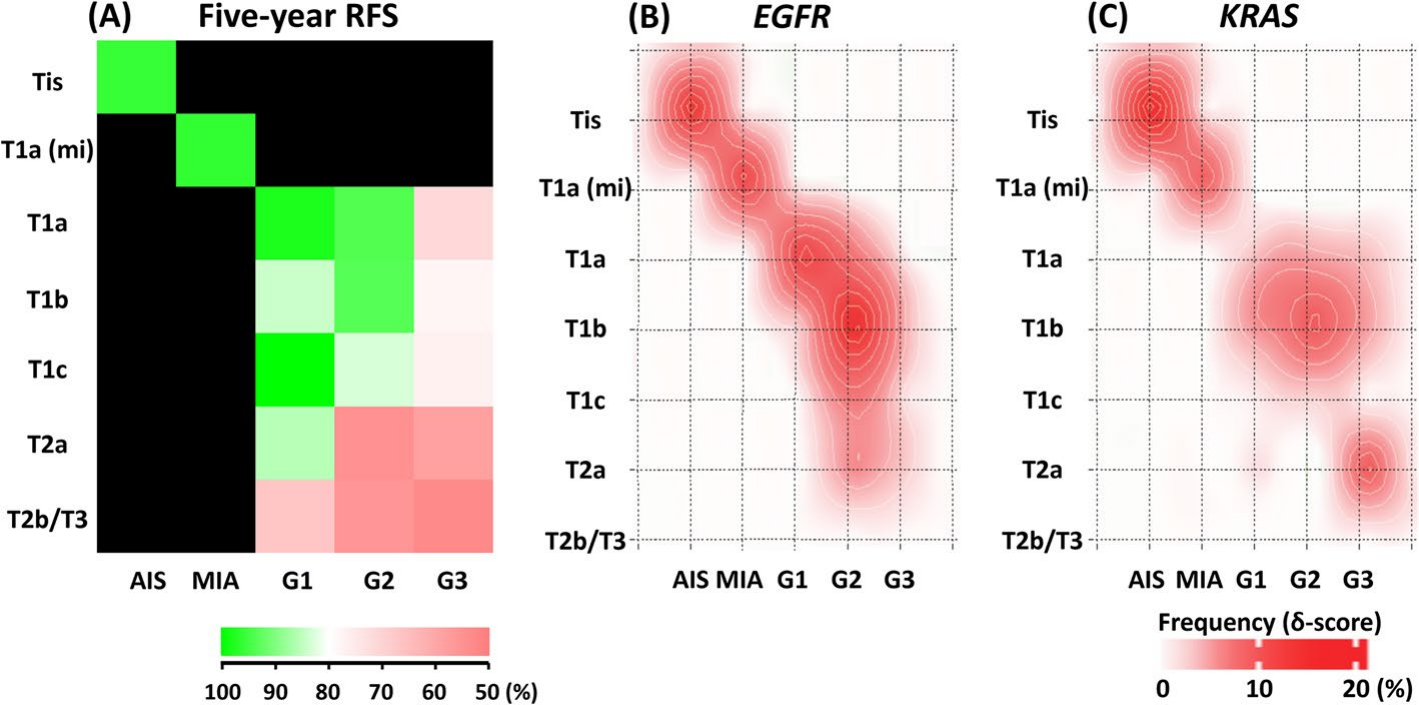
Heatmap showing the five-year RFS rate and Bliss images representing the distribution of cases harboring *EGFR* or *KRAS* mutations. **A** Heatmap showing the five-year RFS rate according to pathological T status or histological grade. **B**, **C** Bliss images representing the distribution of cases harboring *EGFR* (**B**) or *KRAS* mutations **C** according to pathological T status or histological grade. Abbreviations: AIS, adenocarcinoma in situ; G1, grade 1; G2, grade 2; G3, grade 3 MIA, minimally invasive adenocarcinoma; RFS, recurrence-free survival

RFS was lower in cases with more advanced pathological T status or IASLC grading (Fig. 3A). The distribution of cases harboring *EGFR* or *KRAS* mutations changed according to pathological grading. The distribution of *EGFR* mutant cases showed peaks in the AIS, MIA, and T1a/grade 1 cases. In the grade 2 and grade 3 cases, another peak emerged in the grade 2 cases, and no peak was observed in the grade 3 cases (Fig. 3B). Whereas, *KRAS* mutant cases revealed distribution peaks in AIS and MIA cases as well as twin peaks in grade 2 and grade 3 cases (Fig. 3C). In comparison to *EGFR* mutations, cases with more advanced pathological grades were more likely to harbor *KRAS* mutations.

## Discussion

As a predominant cancer-driving genetic variant in lung adenocarcinoma, the prognostic impact of *EGFR* mutation has been discussed. Literature in early phase of the study about prognostic impact of *EGFR* mutation on resected cases reported that cases harboring *EGFR* mutations tend to be accompanied by histological lepidic regions [20, 21] and show better prognosis [3, 22, 23]. On the contrary about the prognosis, several later-coming studies suggested that the risk of recurrence varies by histological or radiological features and that a positive *EGFR* mutation status can be a risk for recurrence after excluding cases with no or little risk of recurrence [1, 2, 24–26].

Contrastingly, studies have consistently claimed that a positive *KRAS* mutation status is related to a poor prognosis [27–30]. Zhang et al. revealed that *KRAS* mutations can be frequently confirmed in never-smokers and that this type of lung cancer shows slow growth [31]. Our study also showed high distribution of *KRAS* mutants in AIS and MIA cases where imply slow tumor growth with good prognosis. Albeit, regarding histological features, *KRAS* mutations are likely to occur in cases with solid components [18, 30, 32]. The latest IASLC grading system defines cases including more than 20% of solid component as high-malignant subtypes [13], and literature suggests that solid component is a risk for recurrence after resection even if it is not the predominant component [33, 34]. In contrast, lepidic lesions disappear during tumor growth [35]. Therefore, as shown in the present study, *EGFR* mutations were more likely to be confirmed in cases with a lower pathological grade, and *KRAS* mutations tended to be positive in cases with a higher pathological grade (Fig. 3). As a result, cases harboring *KRAS* mutations showed higher CRR in IASLC grade 2 and 3 cases.

Literature suggests that the prognostic impact of *EGFR* mutations should be estimated by considering the risk of recurrence based on histological features [1, 2, 24–26]. Even so, we previously predicted that the mere comparison of positive and negative *EGFR* mutation cases can overestimate the recurrence risk of *EGFR* mutation-negative cases if the *EGFR* wild type cohort includes positive *KRAS* mutation cases [4]. Herein, we suggest that the unfavorable prognostic impact of positive *EGFR* mutation status can be more accurately assessed by knowing the *KRAS* mutation status.

Currently, the gene-panel test, which explores genetic variants comprehensively, is widely used in clinical settings [36]. However, comprehensive panel tests are not always used especially in resected cases where cure can be expected by complete resection. In addition, the incidence of other genetic variants is low and their impact on recurrence is less known compared to that of *EGFR* and *KRAS* mutations. Here, we showed that a positive *EGFR* or *KRAS* mutation status is a risk factor for recurrence in resected N0M0 IASLC grade 2 and 3 patients. To evaluate the recurrence risk of *EGFR* mutations, the *KRAS* mutation status should be tested simultaneously.

Our study has some limitations. First, this is a retrospective, single-institution study. Second, we utilized a genetic detection test with high sensitivity (PNA-LNA Clamp PCR for *EGFR*: 0.1% [16], ddPCR for *KRAS*: 0.2% (https://www.bio-rad.com/sites/default/files/webroot/web/pdf/lsr/literature/Bulletin_6679.pdf)); however, not all low frequwnt targetable variants were covered. E709X in Exon 18, insertions in Exon 19 or Exon 20, S768I in Exon 20, or tyrosine kinase inhibitor (TKI)-naive T790M in *EGFR* and somatic mutations in Exon 3 and 4 in *KRAS* were not evaluated. Even if they are rare variants, comprehensive testing might be preferable for achieving a better understanding of the prognostic impact of *EGFR* and *KRAS* mutations. Third, the unfavorable impacts of genetic variants may merely reflect the malignant potential of the tumor invasive status and histological features. Nevertheless, we believe that estimating targetable variants will be advantageous for stratifying cases with similar histology by onco-driver gene status and guiding treatments in adjuvant or post-recurrence cases.

Our study focused on the risk of recurrence and showed no significance in terms of OS. The ultimate aim of stratification is to identify patients with worse prognoses, leading to a longer OS. The post-recurrence prognosis of *EGFR* mutation patients can be prolonged by EGFR TKIs [37–39]. The same is probably also true in patients harboring *KRAS* mutations. In this era, in adjuvant settings or post-recurrence strategies, immune-checkpoint inhibitors should also be considered. Studies simultaneously focusing on the risk of recurrence as well as post-recurrence survival and OS are warranted.

## Conclusion

In conclusion, the risk of recurrence differed according to the tumor invasion status and histological features. The incidence of *EGFR* and *KRAS* mutations also varied according to histological features. Positive *EGFR* and *KRAS* mutation statuses were risk factors for recurrence in resected IASLC grade 2 and 3 patients, and cases with a higher risk of recurrence were more likely to harbor *KRAS* mutations. CRR can be higher in cases harboring *KRAS* mutations than in those with *EGFR* mutations, and the risk of recurrence can be overestimated in *EGFR* mutation-negative cases if *KRAS* mutant cases are simply handled as *EGFR* wild type cases. Determining *EGFR* mutations as a risk factor for recurrence should be considered along with *KRAS* mutation testing.

## Data Availability

The datasets used and/or analyzed during the current study are available from the corresponding author on reasonable request.

## Abbreviations

AIS: Adenocarcinoma *in situ*
CI: Confidence interval
CRR: Cumulative recurrence rate
HR: Hazard ratio
IASLC: International Association for the Study of Lung Cancer
OS: Overall survival
MIA: Minimally invasive adenocarcinoma
RFS: Recurrence-free survival
TKI: Tyrosine kinase inhibitor
